# Coming of age: *EMBO reports* at 25

**DOI:** 10.1038/s44319-025-00572-9

**Published:** 2025-09-08

**Authors:** Bernd Pulverer

**Affiliations:** European Molecular Biology Organization, Meyerhofstrasse, Heidelberg, 69117 Germany

**Keywords:** Science Policy & Publishing

## Abstract

At 25, *EMBO reports* has been part of some major changes in scientific publishing. Time to reflect on the role of a community journal in an era of universal access and discoverability.

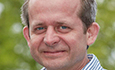

## Teaser

After a quarter century, *EMBO reports* has been part of some major changes in scientific publishing, including the globalization of research, exploding publication numbers, open access, open science and AI. Time to reflect on the role of a community journal in an era of universal access and discoverability.

The scientific journal has a considerably longer history than peer review and the canonical research paper that we now accept as the dominant mechanism for disseminating research findings. Before the dramatic rise of publication rates, the expansion of journal numbers to in excess of 35,000, and the invention of the ‘megajournal’, the purpose of journals was to package a curated set of broadly relevant research articles into browsable monthly issues and disseminate them to a general, scientifically literate audience. With the professionalization of biomedical research and the emergence of sub-disciplines such as molecular biology, genetics, developmental- or cell biology by mid-last century, both the volume and the quality range of journals inexorably expanded, leading to the formalization of the structure of the research paper and the peer review process.

*EMBO reports* was designed to address some of the emerging challenges in publishing with an emphasis on shorter ‘reports’ and a dedicated “Science & Society” section (Gannon, 2025 and Jacobs, 2025). After a quarter century, the journal has evolved beyond these initial innovations and now sits at a crucial interface in the scientific process: on the one hand, E*MBO reports* aims to present research findings that are high-quality and particularly notable to a broad readership. On the other hand, as founding members of the *San Francisco Declaration on Research Assessment* (DORA), its editors select for the highest scientific quality, but not—blindly—for the highest citation potential.

Quality, Impact and Impact factor still tend to be used synonymously with devastating consequences on research assessment and on the research enterprise. *EMBO reports* defines itself as a journal which maintains high selectively for attributes that add value to the scientific community, but not as a journal which rides waves of fashion with an eye primarily on performance metrics or profit. The journal’s editors and referees strive to select for papers that present carefully framed conclusions supported by definitive evidence. The claims must stand out as particularly noteworthy—in this way, we do not only restrict ourselves to conclusions that are unique or novel. In fact, *EMBO reports* explicitly encourages papers reporting null- or refuting-data, or studies reporting first confirmations of important recent discoveries—ideally with orthogonal approaches or an evolutionary complementary angle. We are not beholden to “human relevance” and embrace the whole ecosphere. In conjunction with what Howy Jacobs calls “short and sweet”—that is, papers with notable conclusions not necessarily worked out to a comprehensive mechanistic understanding—*EMBO reports* thus aims to act as buffer to what can appear as a frenzy of funders, hiring committees and journals to prioritize “impact”—erroneously equated to “impact factor”.

In the era of enhanced search functionality and AI, journal selection criteria have to be fundamentally re-evaluated. The original purpose of journals, content enrichment to help individuals assimilate and stay abreast of all key reliable findings, has changed: we can now discover all research findings relevant to our expertise without the aid of journal curation. Nonetheless, I dare to argue that browsing cannot and should not be declared obsolete: if we lose the means to access knowledge that we never expected to see in the first place, then innovation and science will suffer. If young researchers are only ever exposed to results within their existing ‘knowledge horizon’, we run the risk of encouraging derivative science over conceptual breakthroughs.

I am aware that it is already near impossible to stay abreast of research findings even in the increasingly narrow framing of our own specialisms as depth competes ever more with breath. This is where AI will come to the rescue: it will be most powerful in the context of synthesizing knowledge in our own areas of expertise by surveying the space well beyond the corset of “prestige journals”.

In contrast, in the cross-sectorial knowledge space more distant to our own expertise, I continue to see an important role for a broad, high-quality journal such as *EMBO reports*. For this reason, *EMBO reports* will continue to publish papers with notable and robust findings relevant for a broad readership. While we aim to be more inclusive regarding “novelty” and “impact”, we focus in particular on selecting for the most reliable research presented in a manner that is readily reproducible. To achieve this, we require structured methods and encourage protocol posting; we also require that all key findings be posted as “source data” on our companion data repository BioStudies and apply data curation and research integrity screening services to all papers. Thus, the journal is not only open access but it is fully open-science compatible.

As technologies and scientific approaches transcendent field boundaries, the scope of *EMBO reports* has also broadened beyond its molecular-, cell- and developmental origins, to include ecology and evolutionary biology. We are proud that our author base is truly global: the top two countries of origin for research papers are China and the USA.

We are often asked what makes *EMBO reports* unique among EMBO’s publications. It is not the important publication policies EMBO Press implemented, such as transparent peer review, consultative peer review, pre-decision author consultations and scooping protection from preprints. Instead, it is the unique “Science & Society” section, as well as the unique set of selection criteria that goes beyond narrow definitions of “novelty” and “impact”, without any compromise on quality. It is the encouragement of exceptional shorter papers without the need to cross all t’s and dot all i’s mechanistically, without an exclusive focus on conceptual advance and with a species agnostic outlook.

Try it: the journal is still as innovative and fresh as it was in 2000.
